# Automatic delineation of functional lung volumes with ^68^Ga-ventilation/perfusion PET/CT

**DOI:** 10.1186/s13550-017-0332-x

**Published:** 2017-10-10

**Authors:** Pierre-Yves Le Roux, Shankar Siva, Jason Callahan, Yannis Claudic, David Bourhis, Daniel P. Steinfort, Rodney J. Hicks, Michael S. Hofman

**Affiliations:** 1 Cancer Imaging, Peter MacCallum Cancer Centre, 305 Grattan St, Melbourne, 3000 Australia; 2 Nuclear Medicine Department, Brest University Hospital, EA3878 (GETBO) IFR, 148 Brest, France; 3 The Sir Peter MacCallum Department of Oncology, The University of Melbourne, Parkville, Australia; 4 Respiratory Medicine, Peter MacCallum Cancer Centre and Royal Melbourne Hospital, Melbourne, Australia; 5 Service de médecine nucléaire, CHRU de Brest, 29609 Brest CEDEX, France

**Keywords:** V/Q PET/CT, Gallium 68, Regional lung function, Delineation

## Abstract

**Background:**

Functional volumes computed from ^68^Ga-ventilation/perfusion (V/Q) PET/CT, which we have shown to correlate with pulmonary function test parameters (PFTs), have potential diagnostic utility in a variety of clinical applications, including radiotherapy planning. An automatic segmentation method would facilitate delineation of such volumes. The aim of this study was to develop an automated threshold-based approach to delineate functional volumes that best correlates with manual delineation.

Thirty lung cancer patients undergoing both V/Q PET/CT and PFTs were analyzed. Images were acquired following inhalation of Galligas and, subsequently, intravenous administration of ^68^Ga-macroaggreted-albumin (MAA). Using visually defined manual contours as the reference standard, various cutoff values, expressed as a percentage of the maximal pixel value, were applied. The average volume difference and Dice similarity coefficient (DSC) were calculated, measuring the similarity of the automatic segmentation and the reference standard. Pearson’s correlation was also calculated to compare automated volumes with manual volumes, and automated volumes optimized to PFT indices.

**Results:**

For ventilation volumes, mean volume difference was lowest (− 0.4%) using a 15%max threshold with Pearson’s coefficient of 0.71. Applying this cutoff, median DSC was 0.93 (0.87–0.95). Nevertheless, limits of agreement in volume differences were large (− 31.0 and 30.2%) with differences ranging from − 40.4 to + 33.0%.

For perfusion volumes, mean volume difference was lowest and Pearson’s coefficient was highest using a 15%max threshold (3.3% and 0.81, respectively). Applying this cutoff, median DSC was 0.93 (0.88–0.93). Nevertheless, limits of agreement were again large (− 21.1 and 27.8%) with volume differences ranging from − 18.6 to + 35.5%.

Using the 15%max threshold, moderate correlation was demonstrated with FEV1/FVC (*r* = 0.48 and *r* = 0.46 for ventilation and perfusion images, respectively). No correlation was found between other PFT indices.

**Conclusions:**

To automatically delineate functional volumes with ^68^Ga-V/Q PET/CT, the most appropriate cutoff was 15%max for both ventilation and perfusion images. However, using this unique threshold systematically provided unacceptable variability compared to the reference volume and relatively poor correlation with PFT parameters. Accordingly, a visually adapted semi-automatic method is favored, enabling rapid and quantitative delineation of lung functional volumes with ^68^Ga-V/Q PET/CT.

## Background

The severity of physiologic abnormality in the lungs is routinely assessed with pulmonary function tests (PFTs). PFTs are simple, noninvasive, and well-established investigations that provide reliable information about global lung function [1]. However, they do not provide spatial information regarding regional pulmonary distribution, which may be highly heterogeneous, especially in patients with chronic obstructive pulmonary disease (COPD) or other pulmonary diseases such as lung cancer. Establishing a functional map of the regional ventilation and perfusion in the lungs is therefore highly relevant in many clinical situations.

Our group has demonstrated the feasibility of transitioning from conventional single-photon techniques to positron-emission tomography (PET) technology for functional lung imaging [2]. Ventilation imaging can be performed using the same synthesis device as Technegas® (Cyclopharm, Sydney, Australia) by substituting gallium-68 (^68^Ga), a positron-emitting radionuclide, for technetium-99m (^99m^Tc) to produce carbon nanoparticles, which we term “Galligas”. ^68^Ga can also be substituted for ^99m^Tc to label macroaggregated albumin (MAA) for perfusion imaging. This offers a unique opportunity to improve the diagnostic performance of lung imaging, due to the higher sensitivity and spatial resolution, more rapid scan acquisition, and, most importantly, quantitative capability of PET in comparison to conventional scintigraphy [3–6].

In a recent study, we showed a high degree of correlation between visually-defined ^68^Ga-V/Q PET/CT functional lung volumes and PFT parameters [7], suggesting significant potential in management of patients with pulmonary disease. This includes radiotherapy planning for individualizing treatment plans [8, 9] and assessing radiation injury to lungs [10], pre-surgical evaluation of patients undergoing bronchoscopic or surgical lung volume reduction surgery [11, 12], and assessment of pulmonary reserve prior to pulmonary resection surgery [13]. To date, there is no validated method to automatically delineate functional lung volumes with ^68^Ga-V/Q PET/CT. This is a limitation to the diffusion of the technique as a quantitative imaging tool of regional lung function since, while purely manual delineation provides an accurate delineation [7], it is time-consuming and potentially operator-dependent. In addition, manual contouring requires experience in reading V/Q imaging, which may limit its independent use by non-nuclear medicine specialists. In this respect, an automatic segmentation method would facilitate reproducible delineation of these volumes if demonstrated to be reliable. For this purpose, a simple approach would consist in applying a threshold value expressed as a percentage of the maximal value into the lungs. The aim of this study was to assess whether such an automated threshold-based approach can reliably reproduce functional volumes with ^68^Ga V/Q PET/CT using our validated visual method as a reference standard.

## Methods

### Patients

Thirty patients (19 males, 11 females; mean age 65 years, range 46–89 years) were prospectively recruited as part of a study assessing serial change in lung function during radiotherapy (Australian New Zealand Clinical Trial Registry Trial ID 12613000061730). All had locally advanced or inoperable non-small-cell lung cancer and were planned to have radiation therapy with curative intent. All patients underwent PFTs and V/Q PET/CT as part of pretreatment evaluation. The study was approved by the Peter MacCallum Clinical Governance and Ethics Committee, and all patients provided written informed consent. The study design has been extensively described in a previous paper [14].

### ^68^Ga-V/Q PET/CT protocol

All patients underwent a respiratory-gated V/Q PET/CT scan acquired on a GE Discovery 690 PET/CT scanner (GE Medical Systems Milwaukee, WI, USA) as previously described [15]. Ventilation images were acquired following inhalation of Galligas prepared using a Technegas generator (Cyclopharm, Sydney, Australia). Perfusion images were acquired following intravenous administration of approximately 50 MBq of ^68^Ga-MAA. The PET scans were acquired over two bed positions. Respiratory gating of both CT and PET were performed with Varian RPM respiratory tracking system (VarianMedical Systems, Palo Alto, CA). Each PET bed position was acquired for 5 min.

### ^68^Ga-V/Q PET/CT functional volume delineation

#### Whole-lung (WL) volume delineation

The phase of the respiratory cycle during which the PET and CT images were best aligned was chosen for delineation. This was generally in the mid-time expiratory phase of the breathing cycle. The WL was then delineated on the chosen CT scan. An automatic contouring of the lungs based on the Hounsfield unit value was initially performed and then visually adjusted to match normal contours if required.

#### Ventilation and perfusion volume delineation

**Fig. 1:**
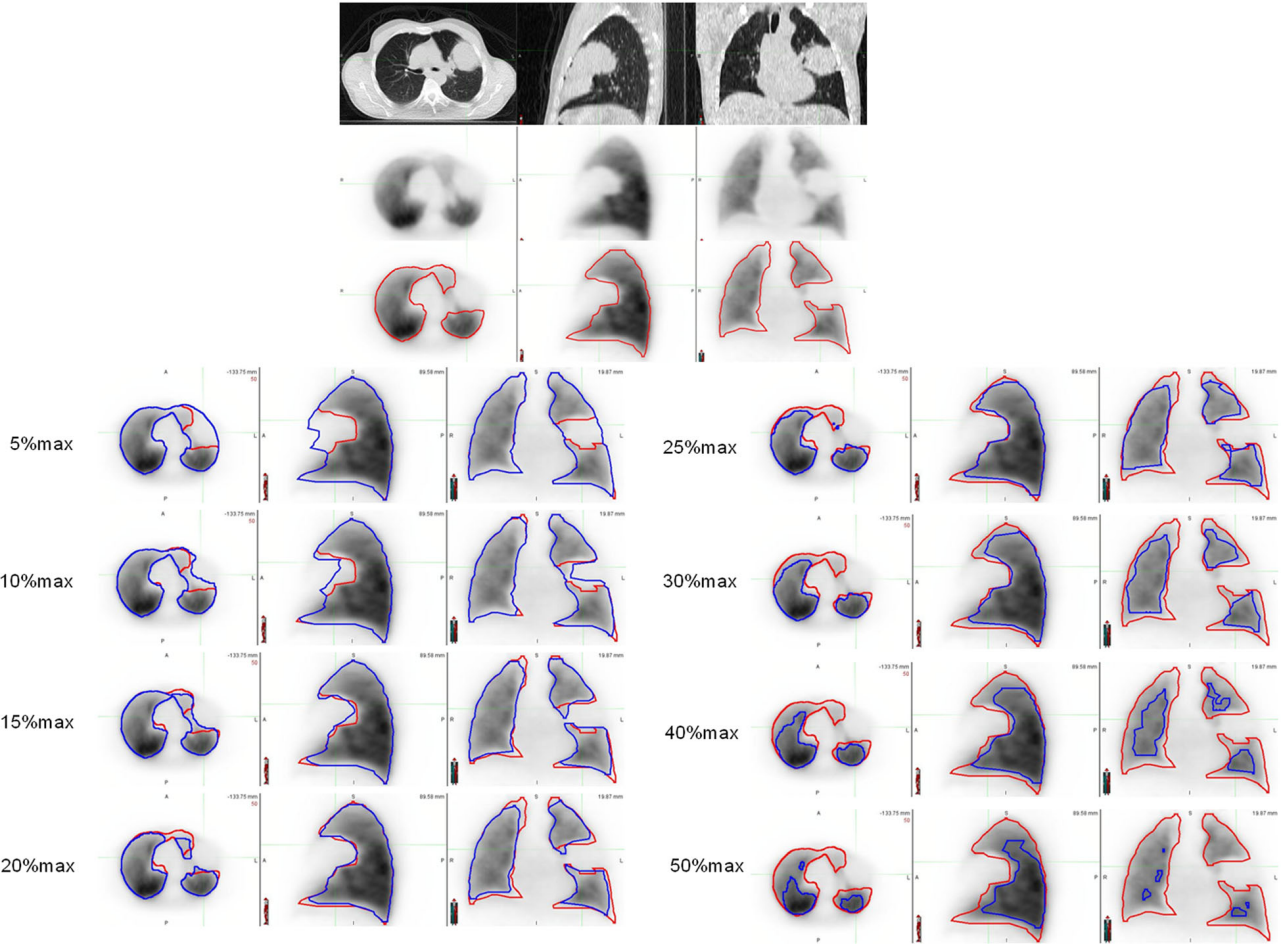
Example of functional perfusion volume delineation. Visually defined manual contour is displayed in red. Automatic contours obtained by applying a cutoff value expressed as a percentage of the maximal value are displayed in blue

For each perfusion and ventilation scan, various cutoff values expressed as a percentage of the maximal pixel value were applied (5, 10, 15, 20, 25, 30, 40, and 50%, respectively) on non-gated PET images. The maximal pixel value was selected by the user in areas without foci of intense uptake (“hot spots”) related to airway deposition. Visually defined manual contours, which we have previously demonstrated to be strongly correlated with PFTs in a prior study [7], were used as reference standard. All volumes were expressed as percentage of the WL. Figure 1 shows an example of perfusion images with the various contours tested.

### Statistical analysis

All statistical tests were carried out using GraphPad Prism 5 (La Jolla, CA, USA). For each threshold, the mean and standard deviation of volume difference between the automatic segmentation and the reference standard were calculated. Dice similarity coefficient (DSC) was calculated, measuring the similarity of volumes. Pearson’s correlation was used to calculate correlation between manual and automatic volumes and between functional volumes and PFT indices. Levels of agreement between the optimal automatic segmentation and the reference standard were analyzed by means of Bland-Altman analysis.

## Results

### Functional ventilation volumes

**Fig. 2:**
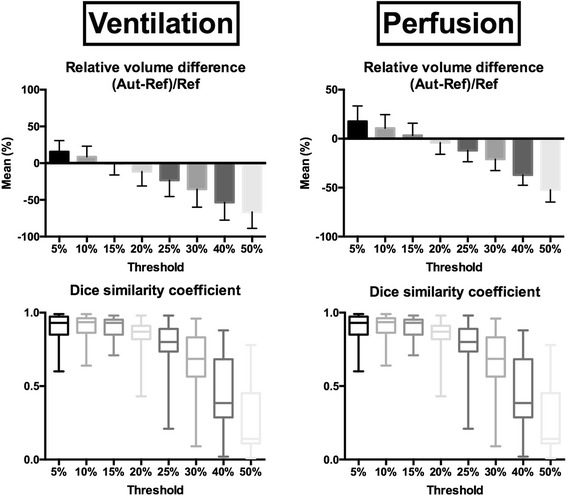
Relative volume difference and Dice similarity coefficient between automatic contours and the reference standard. For volumes, mean and standard deviation (error bars) are displayed. For Dice similarity coefficient, median, interquartile ranges (boxes), and minimal and maximal values are displayed

For ventilation volumes, mean volume difference was lowest using a 15%max threshold (− 0.4 ± 15.6%) with Pearson’s coefficient of 0.71. Applying this cutoff, median Dice similarity coefficient was 0.93 (0.87–0.95) (See Table 1 and Fig. 2).

**Table 1:** Mean, standard deviation, minimum and maximum volume difference between the automatic methods, and the reference standard

		5%	10%	15%	20%	25%	30%	40%	50%
Ventilation	Mean	15.6	8.6	− 0.4	− 11.4	− 23.2	− 35.4	− 53.5	− 66.3
	Std deviation	15.1	14.4	15.6	19.6	22.3	24.5	24.0	22.4
	Minimum	− 0.4	− 12.9	− 40.4	− 66.3	− 80.8	− 87.3	− 90.4	− 92.9
	Maximum	55.7	46.4	33.0	23.2	10.9	1.5	− 12.5	− 24.1
Perfusion	Mean	17.7	10.7	3.3	− 4.1	− 12.1	− 20.9	− 37.0	− 52.2
	Std deviation	15.7	13.4	12.5	11.9	11.4	11.6	10.5	12.56
	Minimum	− 4.4	− 8.2	− 18.6	− 28.4	− 38.3	− 47.7	− 63.4	− 78.2
	Maximum	55.9	46.1	35.5	24.0	11.3	− 0.3	− 18.1	− 29.1

Results are expressed as percentage of the whole lung

**Fig. 3:**
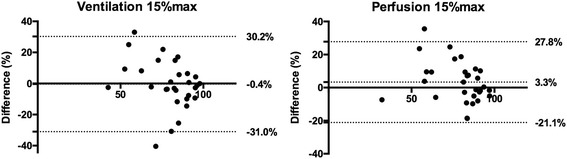
Bland-Altman plot of automatic volumes using the 15%max threshold and the manual contours. The bias and the limits of agreement are displayed in each graphic

While this threshold performed best for the overall group of 30 patients, in individual patients, this was true in only 11 cases. For the remaining 19 patients, the volume difference compared to visual delineation was lowest using another threshold (5%max in 7, 10%max in 2, 20%max in 5, 25%max in 1, and 30%max in 4, respectively). Bland and Altman plot of volumes determined by the reference standard and the 15%max threshold is displayed in Fig. 3. Limits of agreement were − 31.0 and 30.2%, and volume differences ranged from − 40.4 to + 33.0% at this threshold.

### Functional perfusion volumes

For perfusion volumes, mean volume difference was also lowest using a 15%max threshold (3.3 ± 12.5%). Pearson’s coefficient was also highest using this threshold (0.81). Applying this cutoff, median Dice similarity coefficient was 0.93 (0.88–0.93) (See Table 1 and Fig. 2).

However, out of the 30 patients, the lowest volume difference between the automatic and the manual contours was achieved using this 15%max cutoff in only five patients. For the remaining 25 patients, the volume difference was the lowest using another threshold, respectively, 5%max in 3, 10%max in 7, 20%max in 8, 25%max in 5, and 30%max in 2. Bland and Altman plot is displayed in Fig. 3. Limits of agreement were − 21.1 and 27.8% with volume differences ranging from − 18.6 to + 35.5%.

### Comparison of V/Q PET/CT functional volumes with PFT indices

Correlations between PFT parameters and ^68^Ga-V/Q PET/CT functional volumes computed with the manual reference standard [7] and the 15%max fixed threshold are shown in Table 2. Using the automatic contours, moderate correlation was only demonstrated with FEV1/FVC *r* = 0.48 and *r* = 0.46 for ventilation and perfusion images, as compared with *r* = 0.78 and *r* = 0.81 with the reference standard, respectively. No correlation was found between FEV1, DLCO or FVC, and the automatic V/Q PET/CT functional volumes.

**Table 2:** Correlations of PFT parameters and ^68^Ga-V/Q PET/CT functional volumes computed with the manual reference standard and the 15%max fixed threshold

	Reference standard [7]		15%max threshold	
	%WL with normal ventilation	%WL with normal perfusion	%WL with normal ventilation	%WL with normal perfusion
FEV1/FVC	0.78*	0.81*	0.48*	0.46*
FEV1 (%pred)	0.61*	0.62*	0.24	0.25
DLCO (%pred)	0.43*	0.47*	− 0.07	0.00
FVC (%pred)	0.30	0.28	0.03	− 0.01

*indicates *p* value <0.05

## Discussion

In this study, we tested a method to automatically delineate lung functional volumes with ^68^Ga-V/Q PET/CT consisting in applying a fixed threshold value expressed as a percentage of the maximal value. The most appropriate cutoff was 15%max for both ventilation and perfusion images. However, using this unique threshold systematically provided unacceptable difference compared to the reference volume in most individual patients and relatively poor correlation with PFT parameters.

In an initial approach, we looked at global parameters such as Pearson’s coefficients, mean volume difference, and similarity indexes. For both ventilation and perfusion volumes, we found a threshold value (15%max) that provided promising results according to these global parameters. For example, we found with the 15%max threshold for perfusion functional volumes a mean volume difference of − 3.3%, a Pearson’s coefficient of 0.81, and a median Dice similarity coefficient of 0.93.

However, on an individual patient basis, these optimal cutoffs provided, in some patients, wide difference as compared to the visual method. The limits of agreement between the “optimal” automatic volume and the manual volume were large, − 31.0 and 30.2% for ventilation images and − 21.1 and 27.8% for perfusion images, respectively. In addition, the range of volume difference was up to 40.4% for ventilation images and 35.5% of WL for perfusion images. This appears unacceptably wide for a quantitative imaging tool of regional lung function. This was confirmed by assessing correlation with PFT parameters. Indeed, correlation was only observed with FEV1/FVC and was much lower as compared with the manual segmentation. Using an automatic method of delineation consisting in applying the same threshold for all patients is therefore inaccurate and inappropriate for the determination of lung functional volumes with ^68^Ga-V/Q PET/CT.

On the other hand, applying a threshold value expressed as a percentage of the maximal value retains several advantages as compared with the manual method. First, it allows a much more rapid delineation of contours. Second, it may allow a more accurate delineation of small areas of lung dysfunction, especially in patients with heterogeneous disease. Finally, it allows an objective and reproducible description of how the contours were obtained. Accordingly, a threshold expressed as a percentage of the maximal value but visually adapted to an individual patient may be an interesting compromise. The delineation process should start with the 15%max threshold. Based on a visual analysis, lower (e.g., 10%max, 5%max, …) or higher (20%max, 25%max, …) thresholds could then be tried to determine which one provides the most representative of functional volumes. Of note, the range of threshold values was relatively small in our series, from 5%max to 30%max, limiting the number of thresholds needing to be tested.

The main limitation of our delineation method is that it essentially relies on the determination of the maximal value, which then determines the cutoff value. Especially for ventilation images, the presence of focal tracer accumulation due to airway deposition may hamper the determination of the maximal value [16]. In our study, the maximal value was determined excluding such foci, but this process may be somewhat difficult in patients with very heterogeneous lung disease. Future research may focus on other reference value to determine the cutoff. An approach based on an absolute quantification, especially for perfusion image, may also become a reality with PET technology. Finally, the automatic delineation was applied to the whole lung volumes. In scans with an important physiological anterior-posterior gradient, an automatic segmentation may exclude the anterior part of the lungs while it appears to be functional. In that respect, a delineation based on a segmental approach may be of interest.

Another possible limitation of our study is the choice of the visual delineation as the reference standard. Although we have demonstrated this to be correlated with pulmonary function test parameters, the variability in manual delineation may have added to the variability. In addition, we performed gated acquisition, which may limit the generalization of the results to non-gated ^68^Ga-V/Q PET/CT. However, the mid-time expiratory phase of the breathing cycle that was chosen for most of patients is the phase that is usually imaged without gated acquisition.

PET technology offers many additional advantages. It is a noninvasive modality that does not rely on patient effort, except the need to breathe the radioactive gas for a few seconds and to lie relatively still on the PET/CT camera bed during the acquisition time. The acquisition time is low, about 15–20 min with our protocol, and could be reduced due to the high sensitivity of PET technology. There are no known contraindications or acute side effects associated with the radiotracers. The majority of nuclear medicine physicians are now familiar with tomographic images for V/Q scan interpretation [17], enabling rapid familiarization with this new technique. The effective radiation dose of the scan is low, approximately 2 mSv for the PET acquisition plus an additional 1–2 mSv for the low-dose CT component, equivalent to the dose of V/Q SPECT/CT. Finally, ^68^Ga is produced by an on-site generator enabling on-demand availability similar to ^99m^Tc but with a longer shelf life of 9–12 months versus 1–2 weeks for ^99m^Tc generator. The ^68^Ga generator is increasingly available owing to its use for neuroendocrine and prostate cancer imaging [18, 19]. With PET/CT and ^68^Ga becoming increasingly available, we envisage that widespread adoption of V/Q PET/CT could become a reality.

## Conclusions

Using a fixed threshold value for functional lung volume delineation provided unacceptably wide differences of volumes in a significant proportion of patients and much lower correlation with PFT parameters compared to a previously validated manual technique. Therefore, a visually adapted, semi-automatic method is favored for quantitative delineation of functional lung volumes with ^68^Ga-V/Q PET/CT in order to facilitate both accuracy and ease of analysis.
